# The Impact of Health Care Reform Since 2009 on the Efficiency of Primary Health Services: A Provincial Panel Data Study in China

**DOI:** 10.3389/fpubh.2021.735654

**Published:** 2021-10-22

**Authors:** Chaoyang Yan, Hui Liao, Ying Ma, Jing Wang

**Affiliations:** ^1^Department of Health Management, School of Medicine and Health Management, Tongji Medical College, Huazhong University of Science and Technology, Wuhan, China; ^2^The Key Research Institute of Humanities and Social Science of Hubei Province, Huazhong University of Science and Technology, Wuhan, China; ^3^Health Poverty Alleviation Center, Institute for Poverty Reduction and Development, Huazhong University of Science and Technology, Wuhan, China

**Keywords:** primary health care, efficiency, reform, panel data study, health system

## Abstract

**Background:** Primary health care (PHC) is an important part of health systems in the world and in China. To improve the efficiency of PHC institutions (PHCIs), many countries have implemented reforms, including China's health care reform since 2009. This study aims to evaluate the impact of this reform on the efficiency of PHCIs from the perspective of the whole health system.

**Methods:** Data were collected from China Health Statistical Yearbooks and China Statistical Yearbooks published from 2005 to 2019. By taking the number of beds, health technicians and PHCIs as inputs and the proportion of diagnosis, treatment and admission in PHCIs as outputs, Malmquist DEA was used to evaluate the efficiency change of PHCIs, and panel data regression was performed to analyze the impact of the reform and other factors on such efficiency. The interaction between reform and economic level was also estimated.

**Results:** The MPI in Beijing, Tianjin, Shanghai, Hunan, and Guangdong improved after the reform. The efficiency improvement in Beijing, Tianjin and Shanghai is mainly reflected in the growth of TC, whereas the efficiency improvement in Guangdong and Hunan is mainly reflected in the growth of EC. Meanwhile, the EC and TC in Hebei, Heilongjiang, Shandong, and other provinces deteriorated. The deterioration of MPI in Shanxi, Inner Mongolia and Jilin was mainly attributed to EC. while the deterioration of MPI in Liaoning, Anhui, and Fujian provinces is mainly attributed to TC. Since 2009, the reform exerted a negative impact on MPI (β = −0.06; *P* < 0.01), TC (β = −0.048; *P* < 0.01) and EC (β = −0.03; *P* < 0.01). And such negative impact was weaker in economically developed areas (β = 0.076; *P* < 0.01).

**Conclusions:** Attention should be paid to future reforms: China should continue investing in PHCIs, establish a structurally integrated and functionally complementary delivery system and promote the coordination of reform policies to avoid the adverse impacts of other reform policies on PHCIs.

## Background

### Role of PHC in Health System

Primary health care (PHC) has been widely recognized as a crucial part of the overall health service system, and continuously improving its efficiency is essential to achieve universal health coverage. In terms of population health outcomes, a comprehensive and high-quality PHC can meet the majority of the health service needs of people throughout their lifetime (1–3). Maximizing PHC efficiency does not only reduce the wastage of health resources and realize a sustainable development of the health system but also guarantees positive and long-term health outcomes (4, 5).

Previous studies have mostly measured the efficiency of PHCIs from the input–output perspective and analyzed external factors. On the one hand, the relationship between input and output is studied quantitatively. The improvement in health outcomes is related to increased inputs from PHCIs (6–8). On the other hand, data envelopment analysis (DEA) is commonly used to measure the efficiency of PHCIs. The selected input variables mainly include operating costs and the number of administrative and technical personnel and beds, whereas the output variables mainly include the number of outpatients, referrals, hospitalizations, family services, and preventive health services (9–12). Results of these studies revealed a generally low PHCIs efficiency and large efficiency differences across regions. Many studies have pointed out that the population size within the jurisdiction of a health institution serves as the main basis for the allocation and utilization of health resources in PHCIs (13, 14). The aging population trend accelerates the transformation of the service mode of PHCIs to enhance their service efficiency (15, 16). In the process of urbanization, the changes in health service demand and the construction of a health system may affect the utilization of primary health services (17). Macroeconomic development promotes investment in PHCIs, alleviates the economic burden of residents and implements innovative reform measures to promote efficiency (18).

Many countries and regions around the world have implemented reforms aimed at improving the service efficiency of PHCIs. For instance, Singapore implemented a regional health system that aims to promote the integration of different levels of medical systems (19). Meanwhile, the State of Maryland in the US implemented a patient-centered medical home model aimed at improving the efficiency of PHCIs and reducing costs and later proved that this model can improve health outcomes and increase cost savings (20). Moreover, Reforming PHCIs has always been an important goal of China's health system reform.

### Reform of PHC in China

China's health system mainly consists of three-level health service networks in rural areas (village clinics, township hospitals, and county-level hospitals) and two-level health service networks in urban areas (community health service centers and hospitals). In the 1960s and 1970s, relying on the rural collective economy, cooperative medical care, barefoot doctors and the three-level health service network constituted the principle of primary health service (21). It actively promoted people's health and was even praised by the WHO (22–24). In 2003, China implemented a new rural cooperative medical system that consolidates its disintegrating cooperative medical system by expanding its financing scale. However, relevant studies have pointed out that this new system has not fundamentally solved the challenge being faced by China's existing health systems (25).

In 2009, China implemented a comprehensive and relatively thorough health care reform that initially had five priorities, namely, to expand the coverage of medical insurance, to establish an essential drug system, to improve the ability of PHCIs, to promote public health services and to strengthen the reform of public hospitals (26). Among these priorities, strengthening PHC to achieve a universal coverage of basic medical services is an important goal of the reform. During the first 3 years of the reform, the government invested $230 billion, of which about 44% was allocated to PHCIs to provide primary health services (27).

Some studies have analyzed the impact of China's medical reform since 2009 on the efficiency of PHCIs. For instance, Kaili Zhong et al. pointed out that the health resources in the rural PHCIs of Hunan Province increased after the implementation of this reform, but the PHCIs in most counties remained ineffective (5). Yao Leng et al. found that the quantity of health resources and the service efficiency of PHCIs significantly increased after the reform, but the change in management was not obvious (28).

However, the indicators used in these studies were the absolute level of service, and no research has examined the longitudinal changes in the level of service counts of PHCIs relative to that of the entire health service delivery system. Moreover, the differences in the economic development across Chinese provinces are relatively large. Whether the 2009 reform has significantly different effects on the efficiency of PHCIs in regions with varying levels of economic development has not yet been empirically analyzed.

The relative proportion of service counts in PHCIs can objectively and accurately reflect the changes in the capacity and status of PHCIs in the entire health service system. Data from 2005 to 2017 show that the absolute number of outpatients and inpatients in China's PHCIs is growing, but their proportion in the health system exhibits a downward trend compared with tertiary hospitals (29). In addition, the utilization of health services will naturally increase along with improvements of people's ability to pay and their health care awareness. Therefore, this relatively natural service growth may overstate the efficiency of PHCIs and the impact of the reform.

The purpose of this study was (1) to evaluate the changes in the efficiency of PHCIs across various provinces of China before and after the reform since 2009 and to determine the impact of the reform on such efficiency and (2) to analyze whether the relationship between reform and efficiency is significantly differs across provinces with varying economic development levels.

## Methods

### Data Sources

The data used in the study were collected from the China Statistical Yearbooks and China Health Statistical Yearbooks published between 2006 and 2019. These yearbooks provide statistical data on the national and provincial economic, social and health services of China over the past 20 years. These data can be downloaded from public official websites. Under a strict statistical system, the data collection for each province or county is compulsory and is performed by statistical bureaus at all levels. Therefore, no missing data were reported for all variables, provinces and counties. The data used in this study were from the longitudinal data of 2006–2019 of the above two databases.

### Variables and Definitions

In terms of inputs, the main variables selected in the study were the number of beds, health technicians (HTs), and PHCIs per thousand population. These indicators have always been the focus of the policy-making agencies, thereby validating the appropriateness and policy significance of the selected indicators. Previous studies have also used these indicators to evaluate service efficiency (13).

As for the outputs, the number of outpatients and inpatients represent the ability of health institutions to diagnose and treat common and relatively complex diseases. These indicators have also been the focus of policies. The ratio of output in PHCIs to the entire health system was used as another output in this study given its ability to reflect changes in the capabilities of PHCIs in the entire health system. This ratio was selected in line with the objective of increasing the PHC utilization rate in China as emphasized by the 2009 reform. The number of diagnosis and treatments in township hospitals (DToTH), community health service centers (stations) (DToCHC) and hospitals (DToH) and the number of admissions in township hospitals (AoTH), community health service centers (stations) (AoCHC), and hospitals (AoH) were also treated as outputs. The proportion of diagnosis and treatments (PoDT) and the proportion of inpatients (PoIs) in PHCIs were computed as:


$$\begin{array}{l} \text{PoDT}=\frac{\text{DToTH}+\text{DToCHC}}{\text{DToTH}+\text{DToCHC}+\text{DToH}}\\ \text{PoIs}=\frac{\text{AoTH}+\text{AoCHC}}{\text{AoTH}+\text{AoCHC}+\text{AoH}} \end{array}$$

In the covariates, per capita GDP, population, old dependency ratio (ODR), and urbanization rate were used as continuous variables that represent the economic level, total population, aging degree, and urbanization level of a region, respectively. GDP per capita and population were treated logarithmically to alleviate the heteroscedasticity. The reform was included as a binary variable according to time. Table 1 shows the names, definitions, and codes of variables.

**Table 1:** Definition of input–output and control variables.

	**Variables**	**Definition**
Input	Beds	Number of primary health service beds per 1,000 population
	HTs	Number of health technicians in primary health services per 1,000 population
	PHCIs	Number of primary health facilities [including township hospitals and community health service centers (stations)] per 1,000 population
Output	PoDT	The proportion of number of diagnosis and treatment of PHCIs in the total number of the province
	PoIs	The proportion of inpatients in PHCIs in the total number of inpatients in the province
Control variables	GDP	Per capita GDP (adjusted according to CPI)
	Urbanization rate	Proportion of urban population
	Population	Provincial total population
	ODR	The ratio of the population over 65 to the population between 15 and 64
	Reform	Prior to 2008–2009 (excluding 2008–2009), it was assigned a value of 0, and other years were assigned a value of 1.

### Statistical Method

First, the input–output and external factors of the whole country were calculated as the mean and extremum, respectively, to observe the overall changes. Furthermore, the graphic method was used to directly describe the specific changes in the input and output variables within and between provinces.

Second, Malmquist DEA was used to calculate the efficiency of PHCIs in each province. Malmquist productivity index (MPI), which belongs to the framework of non-parametric DEA, is commonly used to evaluate the vertical productivity of decision-making units. The output orientation was adopted in this work because the input is relatively fixed in a short time. This orientation is also in line with the objective of improving the ability of the health system to use existing health resources. Malmquist productivity index (MPI) was used to calculate the changes in the productivity of PHCIs across various provinces over time, which was further decomposed into efficiency change (EC) and potential technology change (TC). TC indicates that the use of new technologies promotes the movement of efficiency boundaries, hence highlighting the role of innovation (30, 31). EC refers to the “catch-up” effect, which is often related to the influence of the management level (32, 33). A value of MPI or any of its components that is greater than (less than) 1 indicates an improvement (deterioration) in performance over the analysis interval (34).

Finally, the regression method of panel data was performed to analyze the impact of the reform and other factors on the efficiency of PHCIs. A series of tests of panel data modeling was applied to identify the appropriate models to analyze the impact of reform on the efficiency of PHCIs. First, an F-test was used to check the appropriateness of using the pool effect least square method. Under the condition of rejecting the null hypothesis (*p* < 0.05), the Hausmann test was conducted to choose fixed and random effects. Results of the Hausmann test showed that the fixed effect model was more suitable when the null hypothesis is rejected; otherwise, the random effect model was more suitable. In addition, the interaction term of reform and per capita GDP was also added to the appropriate model to check for any significant difference in the impact of the reform on provinces with different economic development levels.

RStudio software was used to draw pictures. DEAP2.1 software was used to calculate MPI, EC, and TC in PHCIs. STATA software 13 was used to manipulate panel data models. Statistical significance was set at *p* < 0.05.

### Ethical Approval

Given that all the data used are publicly available, ethical approval was not required for this study.

## Results

Table 2 shows the changes in the national input–output indicators and external factors from 2005 to 2018. On average, HTs, beds, and PHCIs increased from 2005 to 2018. PoIs increased before the reform but decreased after the reform. The same trend was observed for PoDT, but the change in proportion was smaller than that of PoIs. As far as the range of input–output changes were concerned, the beds and HTs were on the rise, among which beds exhibited a larger fluctuation range. By contrast, the PoDT and PoIs showed a downward trend. In terms of external factors, per capita GDP still rose after adjusting for CPI, and the same trend was observed for population, ODR, and urbanization rate.

**Table 2:** Changes in input–output and external factors of PHC in China before and after the reform in 2009.

**Period**		**Inputs**			**Outputs**			**Other external factors**		
		**HTs**	**Beds**	**PHCIs**	**PoDT**	**PoIs**	**GDP**	**Population**	**ODR**	**Urbanization rate**
2005	Mean	0.669	0.524	0.052	0.361	0.219	14364.8	4139.5	12.23	45.41
	Max	1.123	1.056	0.246	0.513	0.404	46331.2	9380	16.24	89.09
	Min	0.344	0.214	0.013	0.120	0.053	4526.8	277	8.64	26.65
2006	Mean	0.680	0.568	0.057	0.382	0.235	16450.9	4165.5	12.4	46.42
	Max	1.073	1.095	0.242	0.663	0.414	51513.4	9392	18.6	88.7
	Min	0.394	0.258	0.013	0.125	0.049	5185.5	281	8.51	27.46
2007	Mean	0.751	0.611	0.059	0.375	0.268	19216.2	4190.9	12.57	47.35
	Max	1.163	1.015	0.239	0.523	0.475	58063.9	9449	18.32	88.7
	Min	0.489	0.262	0.020	0.186	0.033	6081.8	284	8.88	28.24
2008	Mean	0.811	0.694	0.060	0.395	0.279	20142.9	4220.2	12.76	48.20
	Max	1.198	1.054	0.234	0.526	0.501	58318.6	9544	17.33	88.6
	Min	0.503	0.252	0.025	0.216	0.027	7042.4	287	9.16	22.61
2009	Mean	0.886	0.772	0.061	0.387	0.278	20083.6	4247.1	12.82	49.11
	Max	1.230	1.207	0.229	0.498	0.506	58221.9	9638	17.97	88.6
	Min	0.537	0.268	0.030	0.249	0.028	7385.7	290	9.23	23.8
2010	Mean	0.938	0.827	0.064	0.389	0.252	20602.1	4302.7	11.41	50.95
	Max	1.242	1.320	0.227	0.496	0.466	50837.4	10441	16.17	89.3
	Min	0.566	0.219	0.031	0.246	0.021	8332.3	300	7.22	22.7
2011	Mean	0.971	0.853	0.062	0.374	0.224	21674.6	4323.9	11.52	52.17
	Max	1.292	1.406	0.225	0.467	0.418	50990.2	10505	17.36	89.3
	Min	0.591	0.219	0.033	0.241	0.019	9064.7	303	6.71	22.7
2012	Mean	1.005	0.906	0.062	0.372	0.212	21762.3	4348.0	12.05	53.43
	Max	1.406	1.511	0.221	0.492	0.404	51388.1	10594	18.26	89.3
	Min	0.609	0.229	0.033	0.245	0.017	9590.8	308	7.5	22.75
2013	Mean	1.036	0.926	0.062	0.367	0.197	21725.6	4371.5	12.49	54.45
	Max	1.481	1.544	0.220	0.477	0.415	52052.3	10644	18.62	89.6
	Min	0.667	0.215	0.033	0.231	0.014	9967.5	312	7.23	23.71
2014	Mean	1.056	0.945	0.061	0.355	0.176	21819.8	4395.0	12.95	55.55
	Max	1.408	1.572	0.216	0.477	0.371	53079.4	10724	20.04	89.6
	Min	0.687	0.210	0.032	0.226	0.009	10413.2	318	7.86	25.75
2015	Mean	1.079	0.958	0.061	0.350	0.166	21341.1	4422.2	13.67	56.64
	Max	1.456	1.584	0.213	0.464	0.342	53583.7	10849	18.69	87.6
	Min	0.714	0.203	0.032	0.218	0.008	10534.3	324	8.07	27.74
2016	Mean	1.108	0.967	0.061	0.341	0.160	21397.5	4451.1	14.22	57.85
	Max	1.506	1.601	0.208	0.451	0.325	55946.4	10999	19.79	87.9
	Min	0.727	0.203	0.032	0.211	0.008	10381.8	331	7.01	29.56
2017	Mean	1.152	1.020	0.061	0.340	0.158	21591.9	4478.5	15.08	58.98
	Max	1.521	1.670	0.205	0.445	0.314	57222.5	11169	20.6	87.7
	Min	0.778	0.202	0.032	0.216	0.008	10390.8	337	8.22	30.89
2018	Mean	1.188	1.047	0.061	0.330	0.147	21813.3	4504.9	15.69	59.99
	Max	1.572	1.717	0.201	0.422	0.292	58075.1	11346	22.69	88.1
	Min	0.791	0.222	0.032	0.191	0.008	10799.6	344	8.04	31.14

**Figure 1:**
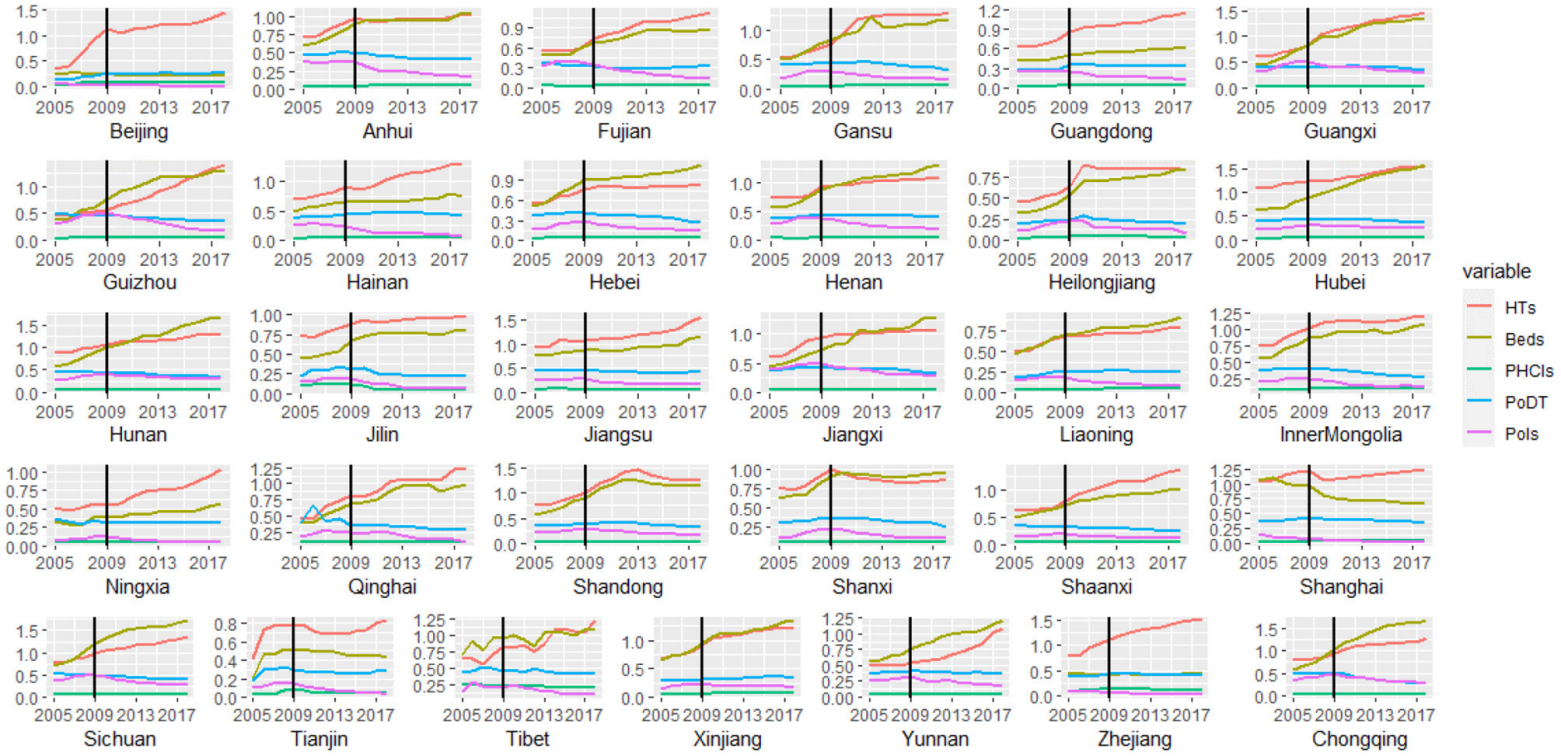
Changes in input and output of China's provinces in 2005–2018.

Figure 1 shows the changes in the input–output of each province from 2005 to 2018. Amongst them, Guangxi, Hebei, Gansu, Guizhou, Inner Mongolia, Qinghai, Jiangxi, Shaanxi, Sichuan, Hunan, Jilin, and Chongqing demonstrated a relatively obvious trend of increasing input and decreasing output. Meanwhile, the input in Anhui, Fujian, Hainan, Henan, Heilongjiang, Liaoning, Tibet, and Yunnan increased, their PoDT did not change significantly, and their PoIs decreased. Hubei, Jiangsu, Ningxia, and Shandong showed an increase in input but no obvious change in output. In Beijing and Zhejiang, only the input HTs increased, whereas the other indicators showed no significant change. In Tianjin and Shanghai, the input beds decreased, while the output PoDT did not change and the PoIs obviously decreased. Differences were observed in the trends for economically developed regions and for those regions with lower economic levels, thereby suggesting that the reform may have an interactive effect with per capita GDP.

**Figure 2:**
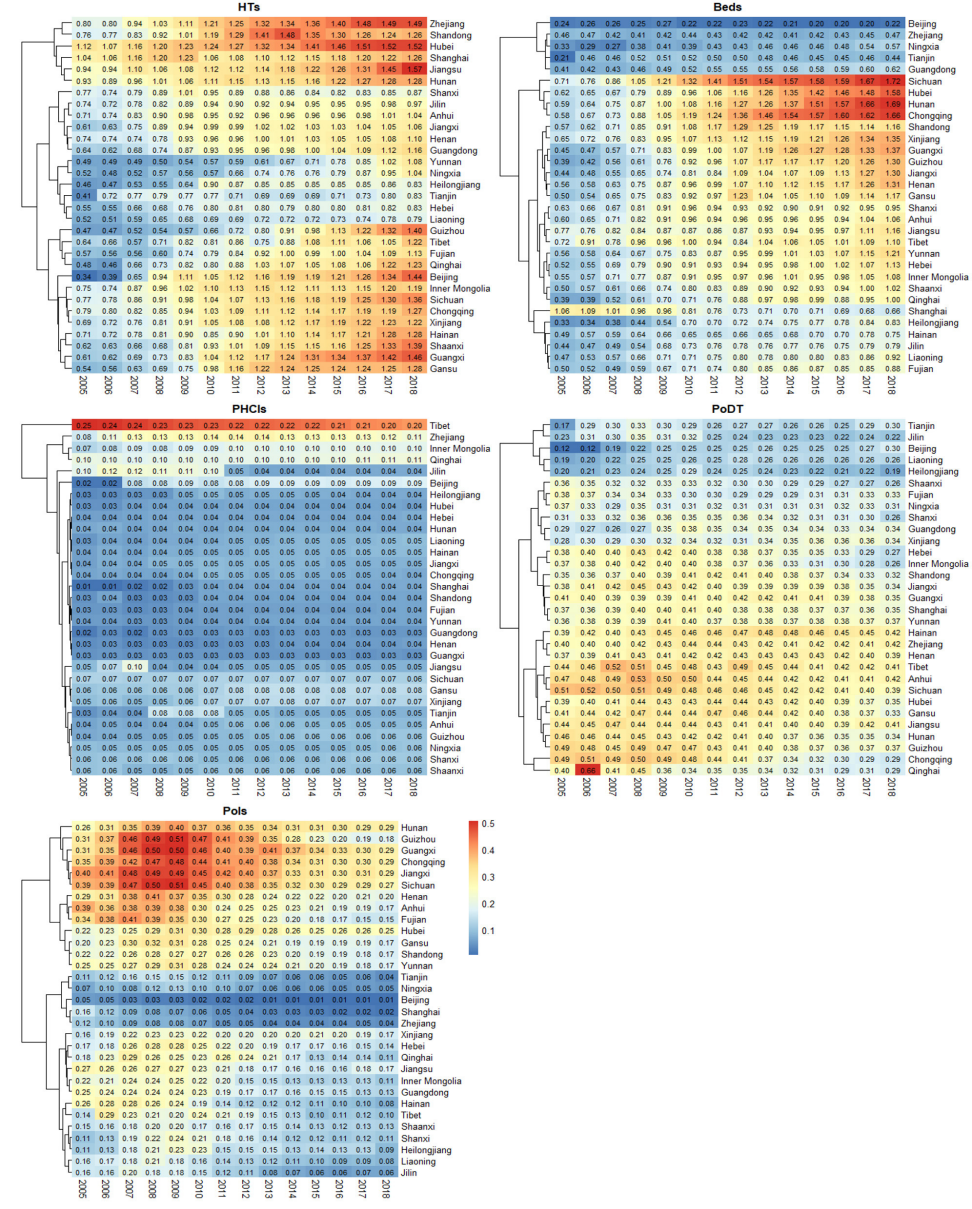
Provincial comparison of input–output indicators in 2005–2018.

Figure 2 shows a horizontal comparison of each input–output indicator across various provinces. Zhejiang, Shandong, Hubei, Jiangsu, and Hunan always maintained high HTs, whilst the HTs in Yunnan, Ningxia, Heilongjiang, Guizhou, Tibet, Qinghai, Beijing, Inner Mongolia, Shaanxi, Guangxi, and Gansu rapidly increased. Beds in Beijing, Shanghai, Zhejiang, Tianjin, and Guangdong were low, whereas those in Sichuan, Hubei, Hunan, Chongqing, Xinjiang, Guangxi, Guizhou, Gansu, Yunnan, and Qinghai grew rapidly. The number of primary health institutions did not change remarkably. Regarding PoDT, Tianjin, Beijing, and Liaoning were always low but showed an upward trend, while the rest of the provinces gradually declined. Amongst these provinces, Sichuan, Chongqing, Guizhou, and Guangxi reported larger declines. With respect to PoIs, Hunan, Guizhou, Guangxi, Chongqing, Jiangxi, Sichuan, Anhui, Fujian, Gansu, Shandong, and Yunnan rose before the reform but declined after the reform, whereas Tianjin, Ningxia, Beijing, Shanghai, and Zhejiang were low. The input–output indicators varied greatly in regions with different economic conditions, which also suggested that the impact of reforms may be affected by economic levels.

Table 3 describes the efficiency in PHCIs in various provinces before and after the reform. At the national level, the EC, TC, and MPI all decreased after the reform, with EC reporting the largest reduction. On average, MPI in Beijing, Tianjin, Shanghai, Hunan, and Guangdong improved after the reform but showed a trend of deterioration in other provinces. The efficiency improvement of Beijing, Tianjin, and Shanghai was mainly the growth of TC, while that of Guangdong and Hunan was mainly the growth of EC. Hebei, Heilongjiang, Shandong, Henan, Chongqing, Guizhou, Yunnan, Tibet, Gansu, and Qinghai showed a deterioration in EC and TC. The deterioration of MPI in Shanxi, Inner Mongolia, Jilin, Jiangsu, Zhejiang, Hainan, and Ningxia was mainly due to EC, whereas the deterioration of MPI in Liaoning, Anhui, Fujian, Jiangxi, Hubei, Guangxi, Sichuan, and Xinjiang was mainly due to TC.

**Table 3:** Efficiency of PHCIs in provinces before and after the reform in 2009.

**Province**	**Before the reform**			**After the reform**			**Difference**		
	**EC**	**TC**	**MPI**	**EC**	**TC**	**MPI**	**EC**	**TC**	**MPI**
Beijing	1.155	0.879	1.020	1.017	1.019	1.036	−0.138	0.14	0.016
Tianjin	1.042	0.933	0.967	1.037	0.945	0.979	−0.005	0.012	0.012
Hebei	1.006	0.977	0.983	1.002	0.941	0.943	−0.004	−0.036	−0.04
Shanxi	1.061	0.933	0.985	1.032	0.938	0.966	−0.029	0.005	−0.019
Inner Mongolia	1.058	0.894	0.937	0.995	0.925	0.920	−0.063	0.031	−0.017
Liaoning	1.008	1.012	1.016	1.047	0.936	0.979	0.039	−0.076	−0.037
Jilin	1.186	0.909	1.056	0.991	0.945	0.938	−0.195	0.036	−0.118
Heilongjiang	1.036	0.928	0.953	0.994	0.913	0.908	−0.042	−0.015	−0.045
Shanghai	1.000	0.860	0.860	0.992	0.967	0.960	−0.008	0.107	0.1
Jiangsu	1.097	0.894	0.978	1.003	0.961	0.963	−0.094	0.067	−0.015
Zhejiang	1.189	0.919	1.007	1.000	0.984	0.984	−0.189	0.065	−0.023
Anhui	1.000	0.967	0.964	1.006	0.939	0.944	0.006	−0.028	−0.02
Fujian	0.980	1.041	1.022	1.000	0.925	0.924	0.02	−0.116	−0.098
Jiangxi	0.976	0.971	0.948	1.008	0.905	0.911	0.032	−0.066	−0.037
Shandong	1.054	0.972	1.026	0.988	0.959	0.947	−0.066	−0.013	−0.079
Henan	1.018	1.024	1.044	1.000	0.954	0.954	−0.018	−0.07	−0.09
Hubei	0.997	0.966	0.962	1.000	0.958	0.958	0.003	−0.008	−0.004
Hunan	0.980	0.949	0.930	1.018	0.933	0.949	0.038	−0.016	0.019
Guangdong	0.931	0.974	0.913	1.028	0.955	0.981	0.097	−0.019	0.068
Guangxi	1.000	1.017	1.017	1.000	0.938	0.938	0	−0.079	−0.079
Hainan	1.038	0.946	0.980	1.012	0.960	0.971	−0.026	0.014	−0.009
Chongqing	1.005	0.974	0.978	1.001	0.924	0.925	−0.004	−0.05	−0.053
Sichuan	0.967	1.022	0.983	1.022	0.909	0.928	0.055	−0.113	−0.055
Guizhou	1.000	1.000	1.000	0.974	0.894	0.870	−0.026	−0.106	−0.13
Yunnan	1.047	0.981	1.026	0.996	0.931	0.927	−0.051	−0.05	−0.099
Tibet	1.110	0.991	1.051	1.014	0.930	0.944	−0.096	−0.061	−0.107
Shaanxi	1.027	0.923	0.940	0.999	0.931	0.929	−0.028	0.008	−0.011
Gansu	1.021	0.940	0.954	0.999	0.921	0.920	−0.022	−0.019	−0.034
Qinghai	1.035	0.959	1.033	0.976	0.917	0.895	−0.059	−0.042	−0.138
Ningxia	1.051	0.914	0.944	0.984	0.957	0.941	−0.067	0.043	−0.003
Xinjiang	1.035	0.966	0.997	1.040	0.925	0.962	0.005	−0.041	−0.035
China	1.036	0.956	0.983	1.006	0.940	0.945	−0.03	−0.016	−0.038

Table 4 shows the relationship amongst MPI, TC, EC, and the reform and external factors in PHCIs. Models (1)–(3) show the impact of the reform on primary health service efficiency in MPI, TC, and EC, respectively. Models (4)–(5) show the interaction of reform and GDP per capita on the efficiency of primary health services in MPI, TC, and EC, respectively. The reform had a significant negative effect on MPI, TC, and EC of PHCIs. The population had a significant positive effect on MPI and TC. It was also found that per capita GDP had a significant interaction between the reform and MPI and TC of efficiency of PHCIs, that is, the adverse impact of reform on MPI and TC in relatively developed areas was less than that in economically underdeveloped areas.

**Table 4:** The impact of reform and external factors on the efficiency of PHCIs.

	**Model (1)**	**Model (2)**	**Model (3)**	**Model (4)**	**Model (5)**	**Model (6)**
	**MPI**	**TC**	**EC**	**MPI**	**TC**	**EC**
lnGDP	−0.094	−0.044	0.032	−0.016	−0.049^**^	0.046^*^
	(0.063)	(0.040)	(0.023)	(0.025)	(0.020)	(0.026)
lnpopulation	0.261^*^	0.393^***^	−0.003	−0.000	0.002	−0.002
	(0.128)	(0.090)	(0.006)	(0.005)	(0.004)	(0.006)
Urbanization rate	0.003	0.002	−0.001	−0.000	−0.000	−0.000
	(0.002)	(0.001)	(0.001)	(0.001)	(0.001)	(0.001)
ODR	−0.002	0.000	−0.001	−0.000	0.001	−0.001
	(0.002)	(0.002)	(0.002)	(0.002)	(0.001)	(0.002)
Reform(ref:0)	−0.060^***^	−0.048^***^	−0.030^***^	−0.764^***^	−0.894^***^	0.193
	(0.012)	(0.013)	(0.010)	(0.186)	(0.148)	(0.193)
Reform interact				0.076^***^	0.092^***^	−0.023
With lnGDP				(0.020)	(0.016)	(0.020)
_cons	−0.332	−1.894^**^	0.789^***^	1.152^***^	1.390^***^	0.651^***^
	(1.322)	(0.833)	(0.192)	(0.218)	(0.173)	(0.226)
Obs.	403	403	403	403	403	403

*Standard errors are in parenthesis*.

*Fixed effect models are used in models 1 and 2 because both the F test and the Hausmann test reject the null hypothesis. Pooled ordinary least squares was applied to models 3–6 because the F test could not reject the null hypothesis*.

*** *p < 0.01*,

** *p < 0.05*,

* *p < 0.1*.

## Discussion

This study measured the efficiency of PHCIs at the provincial level in China and analyzed the impact of the reform since 2009 and potential external factors. Results show that the reform had not achieved the expected goal of promoting the efficiency of PHCIs. Furthermore, there were significant differences among regions with different levels of economic development, and the population size could significantly improve MPI and TC in PHCIs. The relative insufficiency of government investment, the fragmentation of health service delivery system, and the lack of reform coordination could be the factors for these results.

Compared with hospitals, the government's investment in PHCIs is relatively insufficient, which may have contributed to the decline in the efficiency of PHCIs. In terms of infrastructure, from 2010 to 2018 after the reform, the number of primary medical institutions, the number of beds per thousand and the number of health technicians per thousand increased by 4.65, 57.8, and 21% respectively, whereas the indicators of hospitals in the same period increased by 25.3, 77.15, and 38.1% respectively (29). Regarding the growth rate, hospitals were much higher than PHCIs. These gaps in health infrastructure investment indicate that government investment in China remains focused on hospitals. Previous studies showed that township and village clinics provide the correct treatment time for only 38% and 28% patients with TB, respectively (35). And village doctors failed to ask the recommended questions for the diagnosis of the disease 82% of the time for patients with unstable angina (36). Another study pointed out that 26% of patients do not trust community health service centers, which is higher than 6% of hospitals (37). Thus, this poor quality of service and distrust may enforce patients to seek medical services in hospitals.

The fragmented service system may lead to a competition between hospitals and PHCIs for patients, which may prompt the former to offer a larger number of service relative to PHCIs due to their stronger competitiveness. In the early stage of the reform, PHCIs and hospitals were independent institutions. In terms of finance, 90% of the income of hospitals depends on the services they provided (38). The financial income of the hospitals is not affected by PHCIs but is related to patients. Therefore, hospitals have stronger motivation to compete for patients, and their competitiveness is stronger than PHCIs. In addition, in the early stage of the reform, the medical insurance payment system of PHCIs and hospitals was based on fee for service, which triggered a to the impetus of competition amongst institutions for patients. For financial gain, medical institutions tried to retain their patients rather than transfer them to the appropriate institutions. The government introduced new payment methods such as payment by disease type and global budget. However, these payments are mainly based on specific institutions rather than population (38). Thus, a corresponding coordination mechanism is lacking. Recently, the government launched the “Medical Association” of rural three-level service network and “Medical Community” of the urban two-level service network and implemented the global budget within the organization (39, 40). The effect of such implementation still needs a long time to be observed and evaluated accurately.

The fragmented health delivery system is also reflected in the duplication of its functions, which may lead to a homogenization competition amongst different levels of medical institutions. At present, the subsidy for public health services is directly paid by the central and local governments to PHCIs according to the per capita standard (41). As the economic incentives generated by the provision of public health services in PHCIs depend on the number of permanent residents in the jurisdiction, such provision cannot generate additional economic incentives. Moreover, public health services require a longer time to improve the health outcomes of the population. However, on the basis of the number of services provided by PHCIs, it could generate direct benefits and improve the health outcome of patients immediately. Therefore, PHCIs generally pay more attention to medical treatment than prevention and health management. Previous studies showed that the hospital admission rate of hypertension in China is the highest in all OECD countries (42–44). These results indicate that the PHCIs did not achieve the expected goal in delivering basic public health services. These factors aggravated the adverse effects of fragmentation of the health delivery system and the imbalanced resource allocation on PHCIs.

The poor coordination between the reform policies also impedes efficiency improvements in PHCIs. As one of the five priorities of the reform in 2009, the essential drug system aims to reduce the price and promote the accessibility of drugs by publishing a list of commonly used drugs, bidding for the drugs in the list, allocating them to PHCIs, and selling them to patients in accordance with zero mark-up. However, this system brought more adverse effects on PHCIs. Before the implementation of this system, the drug income of PHCIs accounted for more than 50% of its total income, and the zero mark-up for drugs led to a greater decline in the income of institutions and PHCIs physicians (45). In a study of three provinces, the reported income of doctors in Tianjin township hospitals decreased by 15%, whereas that of doctors in Ningxia township hospitals and village clinics decreased by 17% and 22%, respectively (46). Furthermore, after the implementation, those pharmaceutical enterprises with the lowest quotation were often selected for bidding procurement, which also reduced the profits of these pharmaceutical enterprises and limited the stable production capacity of pharmaceutical manufacturers, thereby preventing a timely and effective supply of medicines in PHCIs (46). A study in Beijing pointed out that only 59.7% of traditional Chinese medicine and 49.1% of western medicine in the essential medicine list were stored in community health service centers due to the shortage of drugs (47). The adverse effects of the essential drug system on PHCIs have prompted patients to seek treatment in hospitals.

The more effective reform practices and innovation measures in economically developed areas may explain the regional imbalance of the reform effect. Provinces in China have varying economic development levels and show obvious differences in their PHCIs. Specifically, economically developed areas tend to face less resistance to reform given their excellent health infrastructures. Therefore, these areas are more likely to adopt a series of innovative measures compared with their counterparts. For example, Zhejiang Province has established a full-cycle health management and referral service model for hypertension and diabetes patients. Beijing initiated a comprehensive reform of the separation of medical treatment and medicine, focusing on improving the content and quality of PHC. Shanghai launched diversified care services and cognitive impairment care services for its deeply aging population structure. These measures have a better implementation basis in areas with relatively rich primary health resources and promote the efficiency boundary of PHCIs to move forward to a certain extent, thereby alleviating the adverse impact of the medical reform on the technical efficiency of PHCIs in 2009.

Given that areas with larger populations have greater demand for basic health services, so the continuous accumulation of experience may promote a continuous improvement in the technical level of primary health institutions. Given that PHCIs mainly provide basic medical services for common and frequently-occurring diseases, these services are less difficult than the medical services provided in hospitals. Therefore, relatively more basic medical needs in areas with larger populations may accumulate more experience, and related studies have also pointed out that “learning by doing” is an important way of technological progress. Accumulating long-term experience may promote the advancement of basic medical technology in PHCIs.

To estimate and improve the efficiency of PHCIs, research on the perspective of health service delivery systems by using relative indicators can propose systematic and holistic suggestions for improving the efficiency of the primary health service system. Such work has implications for other developing countries and regions. Moreover, the analysis of the impact of economic level on the relationship between reform and the efficiency of PHCIs can provide insights for different regions to develop highly more accurate intervention measures, which can be used as reference by countries and regions with unbalanced economic development level.

### Limitations

Firstly, village clinics, outpatient departments and school hospitals were excluded from this study. Whilst these institutions are parts of the PHC network, before the reform, the health service data of these institutions were missing from the China Health Statistics Yearbook. To ensure that the data before and after the reform are consistent, this study decided to exclude these institutions from the analysis. Given that previous studies have also pointed out that township hospitals and community health service centers are the main components of PHCIs, excluding these institutions only had little impact on the findings of this work.

Secondly, to calculate the relative value, this study added the hospital service number into the denominator but could not use the service number of the whole PHCIs. Before the reform, there was no service number and summary data about the whole PHC, so this study included the service number of hospitals in the denominator to ensure the comparability of data before and after the reform. Given that hospitals in China are the most important part of both the urban two- and rural three-level health service networks, so this calculation method can accurately reveal relative changes.

Finally, this study did not consider public health indicators as input–output indicators. PHCIs in China provide basic medical and public health services. However, given the unavailability of public health data in various provinces, this study only included medical service indicators.

### Conclusions

The reform since 2009 did not achieve the expected goal of improving the efficiency of PHCIs. In the follow-up reforms, the following measures are necessary: (1) to continue to increase the investment in PHCIs to narrow the gap with hospitals, (2) establish a structurally integrated and functionally complementary delivery system, and (3) avoid the adverse impacts of other policies on the efficiency of primary health services. It should be noted that in terms of the selection of output indicators or the use of methods, the efficiency value calculated in our study is relative efficiency.

## Data Availability Statement

Publicly available datasets were analyzed in this study. This data can be found at: https://data.cnki.net/area/yearbook/single/n2012090077?z=d09; http://www.stats.gov.cn/tjsj/ndsj/.

## Author Contributions

CY conceived the idea and wrote framework and manuscript of the paper. HL and YM collected and sorted the data. JW critically revised the paper. All authors have read and approved the final manuscript.

## Funding

This research was supported by National Natural Science Foundation of China (72074086 and 71673093). The publication of study results was not contingent on the sponsor's approval or censorship of the manuscript.

## Conflict of Interest

The authors declare that the research was conducted in the absence of any commercial or financial relationships that could be construed as a potential conflict of interest.

## Publisher's Note

All claims expressed in this article are solely those of the authors and do not necessarily represent those of their affiliated organizations, or those of the publisher, the editors and the reviewers. Any product that may be evaluated in this article, or claim that may be made by its manufacturer, is not guaranteed or endorsed by the publisher.
